# A Chinese Herbal Medicine, Tokishakuyakusan, Reduces the Worsening of Impairments and Independence after Stroke: A 1-Year Randomized, Controlled Trial

**DOI:** 10.1093/ecam/nep026

**Published:** 2011-04-14

**Authors:** Hirozo Goto, Nobuhiko Satoh, Yoshinori Hayashi, Hiroaki Hikiami, Yutaka Nagata, Ryosuke Obi, Yutaka Shimada

**Affiliations:** ^1^Department of Japanese Oriental Medicine, Graduate School of Medicine and Pharmaceutical Sciences, University of Toyama, Toyama 930-0194, Japan; ^2^Tonami General Hospital, Toyama, Japan; ^3^Yoshimi Hospital, Toyama, Japan

## Abstract

In post-stroke patients, the recurrence of stroke and progression of impairments lead to a bedridden state and dementia. As for their treatments, only anti-hypertension and anti-coagulation therapies to prevent the recurrence of stroke are available. In Asia, post-stroke patients with impairments are often treated with herbal medicine. The present study evaluated the effectiveness of tokishakuyakusan (TS) in improving the impairment and independence in post-stroke patients. Thirty-one post-stroke patients (mean age = 81.4 years) were recruited and enrolled. Participants were randomly assigned to the TS group (*n* = 16) or non-treatment (control) group (*n* = 15) and treated for 12 months. Impairments were assessed using the Stroke Impairment Assessment Set (SIAS). Independence was evaluated using the functional independence measure (FIM). For each outcome measure, mean change was calculated every 3 months. The results were that impairments according to SIAS did not significantly change in the TS group. In contrast, SIAS significantly worsened in the control group. There was a significant difference between the two groups. In each term of SIAS, affected lower extremity scores, abdominal muscle strength, function of visuospatial perception, and so forth. in the TS group were better than those in the control group. Independence according to FIM did not change significantly in the TS group. In contrast, FIM significantly worsened in the control group. There was also a significant difference between the two groups. In conclusion, TS was considered to suppress the impairments of lower limbs and to exert a favorable effect on cerebral function for post-stroke patients.

## 1. Introduction

In post-stroke patients, the recurrence of stroke and progression of impairments lead to a bedridden state and dementia. These are important medical problems in many aging societies. However, as for their treatments, only anti-hypertension and anti-coagulation therapies to prevent the recurrence of stroke are available. In terms of non-drug therapy, only rehabilitation is used for inhibiting the progression of impairments [1, 2].

In East Asia, post-stroke patients with impairments are often treated with herbal medicine [3], acupuncture and moxibustion [4, 5]. Tokishakuyakusan (TS), a traditional Chinese herbal medicine called *Dang-gui-shao-yao-san* in Chinese, is reported to have a neuroendocrine effect [6] and to activate choline acetyltransferase [7]. Recently, the efficacy of TS on cognitive impairment from Alzheimer's disease [8] and improvement of microcirculation in patients with asymptomatic cerebral infarction [9] have been reported clinically. A neuroprotective effect and the improvement of microcirculation are thought to be useful for post-stroke patients. But there has been no report on the long-term effect of TS for post-stroke patients.

The aim of this study was to determine whether TS would improve the impairments and independence of post-stroke patients over a 12-month period in a randomized, controlled trial.

## 2. Methods

### 2.1. Participants

Subjects were recruited from two long-term-care facilities located in Toyama prefecture, Japan. The diagnosis of post-stroke was made on the basis of a history of cerebral bleeding, infarction or subarachnoid hemorrhage as well as having paralysis due to cerebral lesion. The patients had already passed the acute phase of stroke and had been stable for the previous year. At baseline, each patient underwent a uniform evaluation that included medical history, physical and neurological examination, as well as assessment of impairments and independence.

Impairments were assessed using the Stroke Impairment Assessment Set (SIAS), a standardized measure of stroke impairment consisting of the subcategories of motor, tone, sensory, range of motion, pain, trunk function, visuospatial function, speech and unaffected side function, with its high inter-rater reliability having been reported [10, 11]. Independence status was assessed by Functional Independence Measure (FIM), a modality now being used throughout the world [12]. Patients with severe dementia, were bedridden, or had neoplastic or other disease that would likely prevent completion of this study, were excluded. As a result, 31 patients (9 men and 22 women: mean age ± SD, 81.4 ± 8.2 years) were selected. Mean post-stroke duration of all subjects was 68.3 ± 75.3 months. As for original disease, 23 had cerebral infarction, seven had cerebral bleeding and one had subarachnoid hemorrhage. Their complications mainly consisted of hypertension, diabetes and ischemic heart disease, but they were well controlled in this study period. Twelve of the subjects had been using anti-coagulation drugs prior to initiation of this study. All subjects were receiving physio- and ergotherapy.

The study design was approved by the Human Subjects Committee, University of Toyama. All patients provided written informed consent in accordance with ethical guidelines set forth in the 1975 Declaration of Helsinki.

### 2.2. Intervention Protocol

A 12-month randomized, controlled trial was begun between October 2005 and January 2006. Participants were assigned to the TS and control groups using atableof random numbers (Table 1).

TS, which is approved for medical use in Japan, was purchased from Tsumura Co. Ltd (Tokyo, Japan). It consists of six herbs: 4.0 g of Alismatis Rhizoma (*Alisma orientale* JUZEPCZUK), 4.0 g of Paeoniae Radix (*Paeonia lactiflora* PALLAS), 4.0 g of Atractylodis Rhizoma (*Atractylodes lancea* DE CANDOLLE), 4.0 g of Hoelen (*Poria cocos* WOLF), 3.0 g of Cnidii Rhizoma (*Cnidium officinale* MAKINO) and 3.0 g of Angelicae Radix (*Angelica acutiloba* KITAGAWA). The aqueous extract was lyophilized to obtain powder. Lactose (3.5 g) was added to the powder (4.0 g) to make granules (total 7.5 g). Subjects were orally administered TS at 7.5 g/day, 2.5 g three times daily, 30 min after meals for 12 months.

### 2.3. Outcome Determination

The SIAS scores were measured by physiotherapists who did not know which subjects were being treated with TS. The FIM scores were measured by care workers who had no knowledge of who the TS-treated subjects were. Every score was determined five times: at baseline, and every 3 months up to 12 months, the completion of the protocol. Body weights were measured at the same time points.

### 2.4. Statistical Analysis

Changes in SIAS, FIM and body weight from baseline (mean ± SD) were compared by post hoc test with repeated measures of analysis of variance (ANOVA). Other data from baseline to endpoint were also compared by Wilcoxon signed-rank test with Bonferroni correction. *P* <  .05 was required for statistical significance.

## 3. Results

### 3.1. Characteristics of Participants

Only one patient in the TS group withdrew at 6 weeks into the trial because of numbness in his limbs; besides TS, he had been taking amantadine hydrochloride and captopril from a few weeks prior to the start of the trial. As a result, 30 patients completed the protocol. Participants were predominantly female. There were no significant differences in baseline characteristics between the TS and control groups in terms of age, sex, post-stroke interval, original disease or complications. Subjects had a mean SIAS score of 44.3 ± 18.1, a mean FIM score of 64.6 ± 25.6, and a mean body weight of 43.3 ± 7.5 kg. There were no significant differences in baseline scores and body weights between the TS and control groups (Table 2).

### 3.2. Changes in SIAS and Each Item

The mean SIAS score in the TS group did not change through the 12-month trial duration, whereas that in the control group worsened significantly (*P* <  .05), the difference between the two groups being significant (*P* <  .05). At any of the 3-month terms of SIAS, the next scores were significantly different between the two groups. The affected lower extremity scores, which were the knee-extension test and the foot-pat test, did not change from the beginning to 12 months in the TS group, but they decreased significantly in the control group (*P* <  .01). Abdominal muscle strength, a measure of the function of the trunk of the body, improved from the beginning to 12 months in the TS group (*P* <  .05) but it did not change in the control group during the 12 months. The function of visuospatial perception did not change from the beginning to 12 months in the TS group, but it decreased significantly in the control group (*P* <  .05). The muscle strength of unaffected quadriceps, important for maintaining the activity of daily living, did not change in the TS group, but it decreased significantly in the control group (*P* <  .05) (Table 3).

### 3.3. Changes in FIM and Body Weight

The mean FIM score in the TS group did not change during the 12 months, whereas that in the control group worsened significantly (*P* <  .05), the difference between the two groups being significant (*P* <  .01). The body weights of both groups decreased significantly during the 12 months, but there were no significant differences between the two groups (Table 2). In the control group, two subjects had recurrent stroke between 9 and 12 months.

## 4. Discussion

During the long-term period of 12 months, despite the limitation of the small study population, this preliminary and randomized trial suggested that TS inhibited the worsening of impairments and independence in post-stroke patients. As for the method of stroke impairment assessment, several kinds have been developed, such as the Canadian Neurological Scale [13] and NIH Scale [14]. SIAS, used in this study, is the assessment based on the guidelines of the Buffalo Symposium of 1989 [15], and has recently been often used clinically to assess the impairment in post-stroke patients in Japan, as it can assess not only motor functions but also sensory functions and range of motion at the joints [11].

The maximum score on SIAS is 76 points, and participants with SIAS scores around 40 points do not have not complete paralysis, but they do have paresis in affected limbs. Independence was evaluated using FIM, a measurement in use worldwide [12]. The maximum score on FIM is 126 points, and participants with FIM scores around 60 points have a physical state needing slight to middle-level care. Aged participants who have had paresis for 5 or 6 years will show slowly worsening impairments and independence. With the recurrence of stroke, their condition will worsen precipitously.

Recently, by reason of being unable to live alone, many post-stroke patients, whose impairments are of mild or medium grade, are living in long-term care facilities in Japan. In this respect then, participants in this study are not rare cases, and their impairments advance step by step, finally leading to a bedridden state. As this is now a severe medical problem in Japan, any method of treatment that can improve this situation would be more than welcome. There are a few reports concerning the study of the effects of traditional Chinese medicine in post-stroke patients. The effects on aspiration pneumonia [16] and cognitive function [17, 18] of post-stroke patients for the short term were reported. Of course, maintaining function and ability, and prolonging the period of social life are also of major importance. In this regard, TS is one of the most common formulas in oriental traditional medicine and has been used in large numbers of patients for 2000 years. The administration and safety of TS have been clinically established. It is often used for gynecological diseases [19, 20]. There are some reports on climacteric symptoms [21] and improvement of luteal insufficiency [22]. These reports are interesting, as TS has been reported to improve memory disturbance in menopausal rats [7]. In addition, TS is used for dermatological diseases [23] as well as many other medical conditions [24].

**Figure 1:**
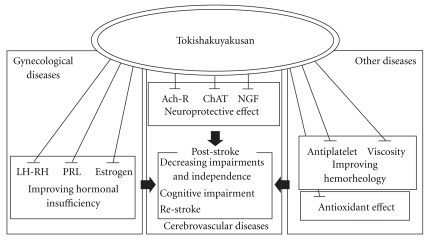
Hypothetical representation of the effects of TS on post-stroke patents. LH-RH: luteum hormone releasing hormone; PRL: prolactin; Ach-R: acetylcholine receptor; ChAT: choline acetyltransferase; NGF: nerve growth factor.

Recently there have been some reports about the treatment of patients with cognitive difficulties with Kampo medicine, as for example, the effect of kihito and BaWei Di Huang Wan on cognitive function [25, 26], and the effect of Yi-Gan San on psychiatric symptoms and sleep structure in dementia patients [27]. But the mechanisms of the functions of these formulas are not yet sufficiently clear. In this study, TS was effective in suppressing impairments of lower limbs and in favorably influencing visuospatial perception. But there are few reports regarding the improvement of muscle strength and paralysis in neurological diseases treated with TS. The mechanisms of these effects are thought to be related to activation of brain function, to have a suppressive effect against re-stroke, and to ameliorate weakness in muscle strength. There are also some reports on TS and the central nervous system. The synthesis and release of neurotransmitters such as acetylcholine, dopamine and norepinephrine have been reported before [6, 7]. Recently, in terms of neuroprotection, the protective effect against amyloid *β*-induced neuronal damage [28] and that after peripheral facial nerve axotomy [29] have been reported. Further, concerning blood circulation, antioxidant and antiplatelet effects [30] and regulation of genes associated with thrombosis [31] have been reported. Clinically, TS has been studied in terms of its efficacy against Alzheimer-type dementia [32], and recently its effect on mild cognitive impairment was reported [8]. Furthermore, we previously demonstrated that TS improved the microcirculation in patients with asymptomatic cerebral infarction. Its mechanisms were believed to improve the hemorheological features of viscosity and erythrocyte deformability and to decrease plasma lipid peroxides [9]. Such favorable effects, which protect neurons and improve cerebral blood circulation, would suppress the worsening of impairments and independence in post-stroke patients. Furthermore, as shown by the improvement of visuospatial perception, TS affected cerebral function, especially the parietal lobe [33]. Another Kampo formula, Chotosan, was also reported to have some activity in frontal lobe function [34]. A hypothetical representation of the effects of TS on post-stroke patients is summarized in Figure 1.

In the present study, subjects were separated into two groups randomly, and they were assessed using SIAS and FIM to easily study impairment and independence. The measurements of SIAS and FIM were used to represent as objective an assessment as possible. The SIAS scores were measured by physiotherapists who were unaware of which subjects were being treated with TS. The FIM scores were measured by similarly blinded care workers. But without the use of placebo, the need for a blind cohort was obviously not satisfied. Although placebo is hard to use in a long-term trial such as the present 1-year term, a well-controlled trial of TS with a larger sample size and the use of comparison drugs will be needed in the future.

In every aging society, keeping the increasing populations of bed-ridden post-stroke patients to a minimum is of major importance, making the findings of this preliminary study, demonstrating that TS could slow deteriorations in impairment and independence in post-stroke subjects, particularly interesting.

## Funding

Grant-in-aid for Funds for Comprehensive Research on Aging and Health from the Japanese Ministry of Health, Labor and Welfare.

## Figures and Tables

**Table 1:** Clinical and demographic characteristics of study participants.

Group	TS group	Control group
Sex (male/female)	4/12	5/10
Age, mean ± SD	81.0 ± 9.4	81.9 ± 7.1
Duration of illness, months, mean ± SD	77.3 ± 86.9	58.6 ± 62.2
Disease		
Cerebral infarction	11	12
Cerebral bleeding	4	3
Subarachnoidal hemorrhage	1	0
Complication		
Hypertension	11	9
Diabetes mellitus	4	5
Ischemic heart disease	1	3
Anti-coagulant drugs	5	7

*Note*: All group comparisons *P* >  .05. SD: standard deviation; TS: Tokishakuyakusan.

**Table 2:** Changes in SIAS, FIM and body weight of TS and control groups.

Outcome	Range	Group	Baseline	3 months	6 months	9 months	12 months	*P*-value^a^
SIAS	0–76	TS	43.6 ± 20.6	45.0 ± 21.5	43.6 ± 20.6	43.6 ± 20.7	43.4 ± 20.5	.044
		Control	43.6 ± 15.9	43.3 ± 15.6	41.9 ± 14.9	41.2 ± 14.7	36.9 ± 16.6*	
FIM	0–126	TS	66.1 ± 29.3	65.5 ± 31.0	66.3 ± 30.6	65.4 ± 31.3	66.2 ± 31.5	.008
		Control	60.8 ± 21.4	58.3 ± 21.4	56.7 ± 20.9	55.8 ± 20.8	49.2 ± 20.7*	
Body weight	(kg)	TS	41.6 ± 7.1	41.7 ± 7.8	41.8 ± 7.4	40.5 ± 7.3	40.1 ± 7.4*	.64
		Control	44.6 ± 7.8	44.3 ± 7.9	43.9 ± 7.6	42.6 ± 7.7	41.9 ± 7.4**	

^a^Comparison of 12-month changes between TS group and control group.

*P <  .05, **P <  .01, comparison from baseline to 12 months in the same group.

**Table 3:** Comparison between TS and control groups on each item of SIAS.

Variable	Range	TS group		Control group		*P*-value^a^
		Baseline	12 months	Baseline	12 months	
Knee-mouse	0–5	2.53 ± 1.92	2.40 ± 0.51	2.13 ± 1.77	1.73 ± 1.75*	.295
Finger-function	0–5	2.33 ± 1.76	2.27 ± 1.87	1.87 ± 1.55	1.40 ± 1.35*	.197
Hip-function	0–5	2.40 ± 1.50	2.27 ± 1.58	2.33 ± 1.40	1.80 ± 1.37**	.262
Knee-extension	0–5	2.27 ± 1.44	2.27 ± 1.39	2.13 ± 1.46	1.40 ± 1.12**	<.001
Foot-pat	0–5	2.33 ± 1.45	2.27 ± 1.39	2.27 ± 1.67	1.73 ± 1.44**	.018
DTR	0–3	1.83 ± 0.98	1.90 ± 0.87	2.10 ± 0.81	2.00 ± 0.78	.106
Tone	0–3	2.10 ± 1.04	1.83 ± 1.10	2.10 ± 0.81	2.00 ± 0.80	.670
Touch	0–3	2.30 ± 0.75	2.13 ± 0.74	2.17 ± 0.77	1.83 ± 0.92	.237
Position	0–3	1.87 ± 1.06	1.87 ± 1.13	1.97 ± 0.90	1.77 ± 0.98	.219
Range of motion	0–3	1.87 ± 1.06	1.87 ± 1.13	1.97 ± 0.90	1.76 ± 0.98	.219
Pain	0–3	2.53 ± 0.64	2.47 ± 0.64	2.73 ± 0.46	2.73 ± 0.46	.560
Abdominal MMT	0–3	1.40 ± 1.06	1.67 ± 1.18*	1.33 ± 0.82	1.20 ± 0.86	.004
Verticality	0–3	2.27 ± 0.80	2.13 ± 0.99	2.07 ± 0.59	1.73 ± 0.88*	.359
Visuo-spat. score	0–3	1.67 ± 1.35	1.80 ± 1.42	1.67 ± 0.98	1.33 ± 1.18*	.031
Speech	0–3	2.00 ± 0.93	1.87 ± 0.92	1.73 ± 0.70	1.40 ± 0.74	.681
Unaffected quadriceps	0–3	1.80 ± 0.78	1.87 ± 0.74	1.93 ± 0.70	1.53 ± 0.83*	.022
Unaffected grip power (kg)		8.93 ± 6.20	8.07 ± 6.31	10.7 ± 7.16	8.80 ± 7.67	.671

DTR, deep tender reflexes; MMT, manual muscle testing; Visuo-spat., visuospatial perception.

^a^Comparison of 12-month changes between TS group and control group.

*P <  .05, **P <  .01, Comparison between baseline and at 12 months in the same group.
